# Bis[3,5-difluoro-2-(2-pyrid­yl)phen­yl](picolinato)iridium(III)

**DOI:** 10.1107/S160053680804083X

**Published:** 2008-12-10

**Authors:** Mao-Liang Xu, Guang-Bo Che, Xiu-Ying Li, Qi Xiao

**Affiliations:** a Xi’an Modern Chemistry Research Institute, Xi’an 710065, People’s Republic of China; b Department of Chemistry, Jilin Normal University, Siping 136000, People’s Republic of China

## Abstract

The Ir centre in the title complex, [Ir(C_11_H_6_F_2_N)_2_(C_6_H_4_NO_2_)], is six-coordinated in a slightly distorted octa­hedral IrC_2_N_3_O fashion.

## Related literature

For background to organic light-emitting diodes (OLEDs), see: Cai *et al.* (2008 ▶); Chen *et al.* (2007 ▶); Park *et al.* (2006 ▶). For the synthesis, see: Lamansky *et al.* (2001 ▶);
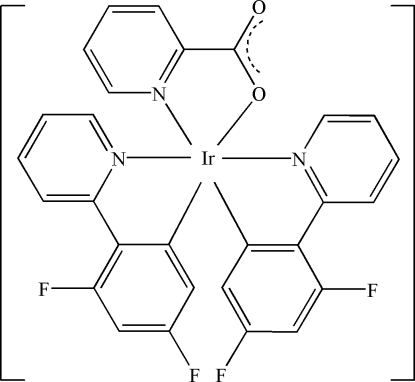


## Experimental

### 

#### Crystal data

[Ir(C_11_H_6_F_2_N)_2_(C_6_H_4_NO_2_)]
*M* *_r_* = 694.64
Orthorhombic, 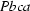

*a* = 16.469 (3) Å
*b* = 14.677 (3) Å
*c* = 19.612 (4) Å
*V* = 4740.3 (16) Å^3^
*Z* = 8
Mo *K*α radiation
μ = 5.70 mm^−1^
*T* = 292 (2) K
0.30 × 0.26 × 0.22 mm

#### Data collection

Rigaku R-AXIS RAPID diffractometer
Absorption correction: multi-scan (*ABSCOR*; Higashi, 1995 ▶) *T*_min_ = 0.203, *T*_max_ = 0.284
43036 measured reflections
5410 independent reflections
4239 reflections with *I* > 2σ(*I*)
*R*_int_ = 0.047

#### Refinement

*R*[*F* ^2^ > 2σ(*F* ^2^)] = 0.034
*wR*(*F* ^2^) = 0.066
*S* = 1.06
5410 reflections
343 parameters
H-atom parameters constrained
Δρ_max_ = 2.31 e Å^−3^
Δρ_min_ = −1.47 e Å^−3^

### 

Data collection: *PROCESS-AUTO* (Rigaku, 1998 ▶); cell refinement: *PROCESS-AUTO*; data reduction: *PROCESS-AUTO*; program(s) used to solve structure: *SHELXS97* (Sheldrick, 2008 ▶); program(s) used to refine structure: *SHELXL97* (Sheldrick, 2008 ▶); molecular graphics: *SHELXTL* (Sheldrick, 2008 ▶); software used to prepare material for publication: *SHELXL97*.

## Supplementary Material

Crystal structure: contains datablocks global, I. DOI: 10.1107/S160053680804083X/bt2823sup1.cif

Structure factors: contains datablocks I. DOI: 10.1107/S160053680804083X/bt2823Isup2.hkl

Additional supplementary materials:  crystallographic information; 3D view; checkCIF report

## Figures and Tables

**Table:** 

C11—Ir	1.997 (5)
C22—Ir	1.993 (4)
N1—Ir	2.041 (4)
N2—Ir	2.045 (4)
N3—Ir	2.138 (4)
Ir—O1	2.152 (3)

**Table:** 

C22—Ir—C11	88.95 (18)
C11—Ir—N1	81.53 (19)
N1—Ir—N2	175.14 (16)
N1—Ir—N3	93.78 (16)
C11—Ir—O1	95.02 (16)
N2—Ir—O1	93.88 (14)

**Fig. 1.**
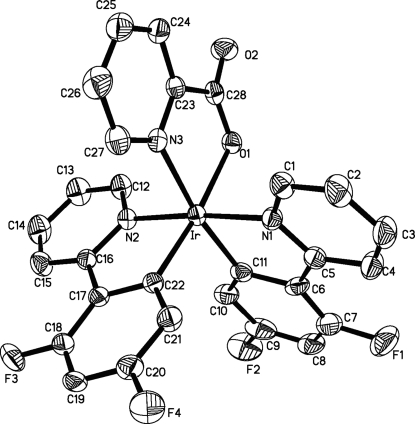
View of the title compound. Displacement ellipsoids are drawn at the 30% probability level (H atoms have been omitted).

**Fig. 2.**
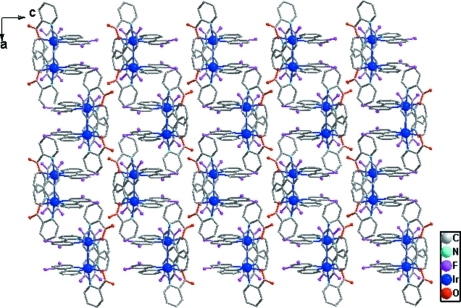
Partial packing diagram of the title compound.

## supplementary crystallographic information

### Comment

In recent decades, the long-lived excited-state and highly efficient solid-state emissions of d^6^ and d^8^ metal complexes have made them of interest as potential components in organic light-emitting diodes (OLEDs) (Chen *et al.*, 2007). Particularly, phosphorescent materials like Ir^3+^ complexes can significantly improve electroluminescent performance because both singlet and triplet excitons can be harvested for light emission, and usually are used as very promising phosphor dyes in OLEDs (Park *et al.*, 2006). Recently, blue organic phosphor such as F_2_Irpic (F = 4,6-difluorophenylpyridinato-N,*C*-2' and pic = picolinate) (I) as a successful cyclometalated Ir^3+^ complex which has been typically doped into host matrices such as tetra-aryl silanes and short conjugation length carbazole derivatives in OLEDs, showing a good quantum efficiency (Cai *et al.*, 2008). In this contribution, we synthesized and investigated crystal structure of F_2_Irpic.

As shown in Fig. 1, each Ir^3+^ cation is in a distorted octahedral coordination geometry, consisting of two chelating cyclometalated F ligands with *cis*-C—C and *trans*-N—N dispositions and one pic ligand. The Ir—O distance being 2.152 Å and Ir—C and Ir—N lengths are in the range of 1.993–1.997 Å and 2.041–2.138 Å, respectively.

### Experimental

The title complex  was obtained in two steps using a standard method (Lamansky *et al.*, 2001) (71% yield based on Ir).

### Refinement

All H atoms on C atoms were positioned geometrically (C—H = 0.93 Å) and refined as riding, with *U*_iso_(H)= 1.2*U*_eq_(C).

### Crystal data

**Table:**

[Ir(C_11_H_6_F_2_N)_2_(C_6_H_4_NO_2_)]	*F*(000) = 2672
*M**_r_* = 694.64	*D*_x_ = 1.947 Mg m^−^^3^
Orthorhombic, *P**b**c**a*	Mo *K*α radiation, λ = 0.71073 Å
Hall symbol: -P 2ac 2ab	Cell parameters from 2859 reflections
*a* = 16.469 (3) Å	θ = 3.0–27.5°
*b* = 14.677 (3) Å	µ = 5.70 mm^−^^1^
*c* = 19.612 (4) Å	*T* = 292 K
*V* = 4740.3 (16) Å^3^	Block, yellow
*Z* = 8	0.30 × 0.26 × 0.22 mm

### Data collection

**Table:**

Rigaku R-AXIS RAPID diffractometer	5410 independent reflections
Radiation source: fine-focus sealed tube	4239 reflections with *I* > 2σ(*I*)
graphite	*R*_int_ = 0.047
Detector resolution: 10.0 pixels mm^-1^	θ_max_ = 27.5°, θ_min_ = 3.0°
ω scan	*h* = −20→21
Absorption correction: multi-scan (*ABSCOR*; Higashi, 1995)	*k* = −19→19
*T*_min_ = 0.203, *T*_max_ = 0.284	*l* = −25→25
43036 measured reflections	

### Refinement

**Table:**

Refinement on *F*^2^	Primary atom site location: structure-invariant direct methods
Least-squares matrix: full	Secondary atom site location: difference Fourier map
*R*[*F*^2^ > 2σ(*F*^2^)] = 0.034	Hydrogen site location: inferred from neighbouring sites
*wR*(*F*^2^) = 0.066	H-atom parameters constrained
*S* = 1.06	*w* = 1/[σ^2^(*F*_o_^2^) + (0.014*P*)^2^ + 17.09*P*] where *P* = (*F*_o_^2^ + 2*F*_c_^2^)/3
5410 reflections	(Δ/σ)_max_ = 0.002
343 parameters	Δρ_max_ = 2.31 e Å^−^^3^
0 restraints	Δρ_min_ = −1.47 e Å^−^^3^

### Special details

**Table:**

Geometry. All e.s.d.'s (except the e.s.d. in the dihedral angle between two l.s. planes) are estimated using the full covariance matrix. The cell e.s.d.'s are taken into account individually in the estimation of e.s.d.'s in distances, angles and torsion angles; correlations between e.s.d.'s in cell parameters are only used when they are defined by crystal symmetry. An approximate (isotropic) treatment of cell e.s.d.'s is used for estimating e.s.d.'s involving l.s. planes.
Refinement. Refinement of *F*^2^ against ALL reflections. The weighted *R*-factor *wR* and goodness of fit *S* are based on *F*^2^, conventional *R*-factors *R* are based on *F*, with *F* set to zero for negative *F*^2^. The threshold expression of *F*^2^ > σ(*F*^2^) is used only for calculating *R*-factors(gt) *etc*. and is not relevant to the choice of reflections for refinement. *R*-factors based on *F*^2^ are statistically about twice as large as those based on *F*, and *R*- factors based on ALL data will be even larger.

### Fractional atomic coordinates and isotropic or equivalent isotropic displacement parameters (Å^2^)

**Table:**

	*x*	*y*	*z*	*U*_iso_*/*U*_eq_	
C1	0.6207 (4)	0.1101 (4)	0.6853 (3)	0.0499 (13)	
H1	0.5661	0.1221	0.6775	0.060*	
C2	0.6411 (4)	0.0328 (4)	0.7211 (3)	0.0669 (17)	
H2	0.6013	−0.0075	0.7359	0.080*	
C3	0.7215 (5)	0.0169 (4)	0.7343 (3)	0.0707 (19)	
H3	0.7368	−0.0343	0.7591	0.085*	
C4	0.7802 (4)	0.0765 (4)	0.7107 (3)	0.0622 (16)	
H4	0.8348	0.0660	0.7200	0.075*	
C5	0.7565 (3)	0.1537 (4)	0.6724 (2)	0.0480 (13)	
C6	0.8089 (3)	0.2221 (4)	0.6425 (3)	0.0473 (13)	
C7	0.8932 (4)	0.2253 (5)	0.6464 (3)	0.0657 (18)	
C8	0.9387 (3)	0.2931 (5)	0.6192 (3)	0.0658 (18)	
H8	0.9949	0.2938	0.6241	0.079*	
C9	0.8993 (4)	0.3605 (5)	0.5842 (3)	0.071 (2)	
C10	0.8160 (3)	0.3619 (4)	0.5770 (3)	0.0542 (15)	
H10	0.7911	0.4092	0.5534	0.065*	
C11	0.7702 (3)	0.2939 (3)	0.6046 (2)	0.0419 (11)	
C12	0.6176 (3)	0.4753 (4)	0.5608 (3)	0.0509 (13)	
H12	0.6167	0.4868	0.6074	0.061*	
C13	0.6061 (4)	0.5465 (4)	0.5168 (3)	0.0661 (17)	
H13	0.5978	0.6053	0.5330	0.079*	
C14	0.6071 (4)	0.5284 (4)	0.4480 (3)	0.0688 (18)	
H14	0.5994	0.5755	0.4169	0.083*	
C15	0.6194 (4)	0.4416 (4)	0.4248 (3)	0.0558 (15)	
H15	0.6196	0.4297	0.3782	0.067*	
C16	0.6316 (3)	0.3711 (3)	0.4712 (2)	0.0385 (11)	
C17	0.6479 (3)	0.2753 (3)	0.4558 (2)	0.0383 (10)	
C18	0.6517 (3)	0.2361 (4)	0.3914 (3)	0.0472 (12)	
C19	0.6645 (3)	0.1461 (4)	0.3795 (3)	0.0518 (14)	
H19	0.6669	0.1222	0.3356	0.062*	
C20	0.6738 (3)	0.0926 (4)	0.4362 (3)	0.0541 (14)	
C21	0.6723 (3)	0.1253 (4)	0.5017 (3)	0.0515 (14)	
H21	0.6804	0.0859	0.5382	0.062*	
C22	0.6587 (3)	0.2175 (3)	0.5135 (2)	0.0357 (9)	
C23	0.4938 (3)	0.3110 (3)	0.6744 (2)	0.0386 (11)	
C24	0.4141 (3)	0.3031 (4)	0.6958 (3)	0.0525 (14)	
H24	0.3964	0.3333	0.7348	0.063*	
C25	0.3613 (4)	0.2499 (4)	0.6585 (3)	0.0628 (17)	
H25	0.3079	0.2422	0.6728	0.075*	
C26	0.3882 (4)	0.2091 (5)	0.6007 (4)	0.0697 (18)	
H26	0.3528	0.1744	0.5744	0.084*	
C27	0.4686 (3)	0.2192 (4)	0.5809 (3)	0.0556 (14)	
H27	0.4864	0.1913	0.5410	0.067*	
C28	0.5564 (3)	0.3678 (3)	0.7132 (2)	0.0430 (12)	
N1	0.6763 (3)	0.1695 (3)	0.6610 (2)	0.0417 (10)	
N2	0.6303 (2)	0.3893 (3)	0.53947 (19)	0.0365 (9)	
N3	0.5214 (2)	0.2683 (3)	0.6182 (2)	0.0391 (9)	
F1	0.9340 (2)	0.1567 (3)	0.6813 (2)	0.0938 (13)	
F2	0.9423 (2)	0.4287 (3)	0.5565 (2)	0.1009 (15)	
F3	0.6422 (2)	0.2901 (3)	0.33525 (15)	0.0727 (10)	
F4	0.6870 (3)	0.0019 (2)	0.4263 (2)	0.0847 (12)	
Ir	0.649485 (11)	0.281026 (12)	0.603051 (9)	0.03453 (6)	
O1	0.6297 (2)	0.3595 (2)	0.69428 (16)	0.0409 (8)	
O2	0.5319 (2)	0.4165 (3)	0.7596 (2)	0.0652 (11)	

### Atomic displacement parameters (Å^2^)

**Table:**

	*U*^11^	*U*^22^	*U*^33^	*U*^12^	*U*^13^	*U*^23^
C1	0.059 (3)	0.041 (3)	0.050 (3)	−0.002 (2)	0.001 (3)	0.007 (3)
C2	0.091 (5)	0.046 (3)	0.064 (4)	0.003 (3)	0.003 (4)	0.016 (3)
C3	0.094 (5)	0.056 (4)	0.061 (4)	0.026 (4)	−0.010 (4)	0.010 (3)
C4	0.067 (4)	0.060 (4)	0.059 (4)	0.021 (3)	−0.011 (3)	0.004 (3)
C5	0.056 (3)	0.051 (3)	0.036 (3)	0.011 (3)	−0.005 (2)	−0.012 (2)
C6	0.033 (2)	0.060 (3)	0.049 (3)	0.008 (2)	−0.003 (2)	−0.024 (3)
C7	0.050 (3)	0.089 (5)	0.057 (4)	0.010 (4)	−0.004 (3)	−0.019 (4)
C8	0.032 (3)	0.098 (5)	0.068 (4)	−0.003 (3)	0.000 (3)	−0.023 (4)
C9	0.050 (4)	0.093 (5)	0.070 (4)	−0.031 (4)	0.022 (3)	−0.023 (4)
C10	0.042 (3)	0.069 (4)	0.052 (3)	−0.005 (3)	0.002 (3)	−0.017 (3)
C11	0.044 (3)	0.045 (3)	0.037 (2)	−0.001 (2)	0.001 (2)	−0.012 (2)
C12	0.067 (4)	0.034 (3)	0.052 (3)	0.001 (3)	−0.009 (3)	0.003 (2)
C13	0.090 (5)	0.038 (3)	0.070 (4)	0.001 (3)	−0.009 (4)	0.001 (3)
C14	0.091 (5)	0.047 (4)	0.068 (4)	0.004 (3)	−0.011 (4)	0.017 (3)
C15	0.070 (4)	0.055 (4)	0.043 (3)	0.002 (3)	0.001 (3)	0.012 (3)
C16	0.035 (3)	0.043 (3)	0.038 (2)	−0.001 (2)	0.000 (2)	0.004 (2)
C17	0.032 (2)	0.045 (3)	0.038 (2)	−0.001 (2)	0.001 (2)	−0.003 (2)
C18	0.037 (2)	0.064 (4)	0.040 (3)	0.001 (3)	0.003 (2)	0.001 (3)
C19	0.042 (3)	0.073 (4)	0.041 (3)	0.007 (3)	−0.003 (2)	−0.024 (3)
C20	0.057 (3)	0.047 (3)	0.058 (4)	0.009 (3)	−0.006 (3)	−0.019 (3)
C21	0.063 (4)	0.038 (3)	0.054 (3)	0.009 (2)	−0.004 (3)	−0.003 (2)
C22	0.035 (2)	0.036 (2)	0.036 (2)	−0.001 (2)	−0.001 (2)	−0.005 (2)
C23	0.045 (3)	0.035 (3)	0.036 (3)	0.002 (2)	−0.001 (2)	0.010 (2)
C24	0.046 (3)	0.062 (4)	0.050 (3)	0.005 (3)	−0.003 (3)	0.011 (3)
C25	0.047 (3)	0.074 (4)	0.068 (4)	−0.011 (3)	−0.001 (3)	0.018 (3)
C26	0.053 (3)	0.077 (5)	0.079 (5)	−0.021 (3)	−0.012 (3)	−0.002 (4)
C27	0.060 (3)	0.054 (3)	0.052 (3)	−0.008 (3)	−0.010 (3)	−0.005 (3)
C28	0.054 (3)	0.038 (3)	0.037 (3)	0.002 (2)	−0.007 (2)	0.003 (2)
N1	0.046 (2)	0.040 (2)	0.039 (2)	0.0068 (19)	−0.0049 (19)	−0.0004 (19)
N2	0.040 (2)	0.032 (2)	0.038 (2)	−0.0019 (16)	−0.0039 (17)	0.0008 (17)
N3	0.038 (2)	0.039 (2)	0.040 (2)	−0.0031 (17)	−0.0062 (17)	0.0059 (18)
F1	0.054 (2)	0.119 (4)	0.108 (3)	0.028 (2)	−0.021 (2)	−0.010 (3)
F2	0.072 (3)	0.116 (4)	0.116 (3)	−0.041 (3)	0.026 (3)	−0.009 (3)
F3	0.094 (3)	0.089 (3)	0.0345 (16)	0.010 (2)	−0.0010 (17)	0.0034 (17)
F4	0.118 (3)	0.051 (2)	0.086 (3)	0.023 (2)	−0.012 (2)	−0.028 (2)
Ir	0.04007 (10)	0.03117 (9)	0.03235 (9)	0.00048 (8)	−0.00413 (8)	−0.00017 (8)
O1	0.044 (2)	0.0416 (19)	0.0376 (17)	−0.0021 (15)	−0.0060 (15)	−0.0040 (15)
O2	0.061 (3)	0.078 (3)	0.057 (2)	0.009 (2)	0.002 (2)	−0.023 (2)

### Geometric parameters (Å, °)

**Table:**

C1—N1	1.351 (6)	C16—N2	1.366 (6)
C1—C2	1.375 (7)	C16—C17	1.463 (7)
C1—H1	0.9300	C17—C18	1.388 (7)
C2—C3	1.370 (9)	C17—C22	1.426 (7)
C2—H2	0.9300	C18—C19	1.359 (8)
C3—C4	1.383 (9)	C18—F3	1.366 (6)
C3—H3	0.9300	C19—C20	1.370 (8)
C4—C5	1.415 (8)	C19—H19	0.9300
C4—H4	0.9300	C20—F4	1.363 (6)
C5—N1	1.359 (7)	C20—C21	1.371 (7)
C5—C6	1.448 (8)	C21—C22	1.391 (7)
C6—C7	1.391 (7)	C21—H21	0.9300
C6—C11	1.439 (7)	C22—Ir	1.993 (4)
C7—C8	1.355 (9)	C23—N3	1.346 (6)
C7—F1	1.391 (8)	C23—C24	1.383 (7)
C8—C9	1.368 (10)	C23—C28	1.528 (7)
C8—H8	0.9300	C24—C25	1.379 (8)
C9—F2	1.341 (7)	C24—H24	0.9300
C9—C10	1.380 (8)	C25—C26	1.356 (9)
C10—C11	1.362 (7)	C25—H25	0.9300
C10—H10	0.9300	C26—C27	1.388 (8)
C11—Ir	1.997 (5)	C26—H26	0.9300
C12—N2	1.345 (6)	C27—N3	1.347 (6)
C12—C13	1.368 (8)	C27—H27	0.9300
C12—H12	0.9300	C28—O2	1.226 (6)
C13—C14	1.377 (9)	C28—O1	1.269 (6)
C13—H13	0.9300	N1—Ir	2.041 (4)
C14—C15	1.367 (8)	N2—Ir	2.045 (4)
C14—H14	0.9300	N3—Ir	2.138 (4)
C15—C16	1.392 (7)	Ir—O1	2.152 (3)
C15—H15	0.9300		
			
N1—C1—C2	123.2 (6)	C18—C19—H19	122.1
N1—C1—H1	118.4	C20—C19—H19	122.1
C2—C1—H1	118.4	F4—C20—C19	117.5 (5)
C3—C2—C1	118.2 (6)	F4—C20—C21	118.6 (5)
C3—C2—H2	120.9	C19—C20—C21	123.9 (5)
C1—C2—H2	120.9	C20—C21—C22	120.0 (5)
C2—C3—C4	120.3 (6)	C20—C21—H21	120.0
C2—C3—H3	119.9	C22—C21—H21	120.0
C4—C3—H3	119.9	C21—C22—C17	117.8 (4)
C3—C4—C5	119.4 (6)	C21—C22—Ir	127.9 (4)
C3—C4—H4	120.3	C17—C22—Ir	114.3 (3)
C5—C4—H4	120.3	N3—C23—C24	122.0 (5)
N1—C5—C4	119.5 (6)	N3—C23—C28	115.7 (4)
N1—C5—C6	113.3 (5)	C24—C23—C28	122.3 (5)
C4—C5—C6	127.3 (5)	C25—C24—C23	119.0 (6)
C7—C6—C11	116.5 (6)	C25—C24—H24	120.5
C7—C6—C5	126.6 (6)	C23—C24—H24	120.5
C11—C6—C5	116.9 (4)	C26—C25—C24	119.2 (6)
C8—C7—F1	117.3 (6)	C26—C25—H25	120.4
C8—C7—C6	123.7 (7)	C24—C25—H25	120.4
F1—C7—C6	119.0 (7)	C25—C26—C27	119.9 (6)
C7—C8—C9	117.8 (6)	C25—C26—H26	120.1
C7—C8—H8	121.1	C27—C26—H26	120.1
C9—C8—H8	121.1	N3—C27—C26	121.4 (6)
F2—C9—C8	119.5 (6)	N3—C27—H27	119.3
F2—C9—C10	118.2 (7)	C26—C27—H27	119.3
C8—C9—C10	122.3 (6)	O2—C28—O1	125.8 (5)
C11—C10—C9	120.0 (6)	O2—C28—C23	117.8 (5)
C11—C10—H10	120.0	O1—C28—C23	116.4 (4)
C9—C10—H10	120.0	C1—N1—C5	119.3 (5)
C10—C11—C6	119.7 (5)	C1—N1—Ir	124.6 (4)
C10—C11—Ir	127.9 (4)	C5—N1—Ir	116.0 (4)
C6—C11—Ir	112.3 (4)	C12—N2—C16	119.4 (4)
N2—C12—C13	122.9 (5)	C12—N2—Ir	124.3 (3)
N2—C12—H12	118.6	C16—N2—Ir	116.3 (3)
C13—C12—H12	118.6	C23—N3—C27	118.5 (4)
C12—C13—C14	118.0 (6)	C23—N3—Ir	114.0 (3)
C12—C13—H13	121.0	C27—N3—Ir	127.4 (4)
C14—C13—H13	121.0	C22—Ir—C11	88.95 (18)
C15—C14—C13	120.5 (6)	C22—Ir—N1	95.68 (18)
C15—C14—H14	119.8	C11—Ir—N1	81.53 (19)
C13—C14—H14	119.8	C22—Ir—N2	80.69 (18)
C14—C15—C16	119.8 (6)	C11—Ir—N2	95.13 (18)
C14—C15—H15	120.1	N1—Ir—N2	175.14 (16)
C16—C15—H15	120.1	C22—Ir—N3	99.04 (17)
N2—C16—C15	119.5 (5)	C11—Ir—N3	171.13 (17)
N2—C16—C17	113.1 (4)	N1—Ir—N3	93.78 (16)
C15—C16—C17	127.3 (5)	N2—Ir—N3	89.99 (15)
C18—C17—C22	118.1 (4)	C22—Ir—O1	173.55 (16)
C18—C17—C16	126.4 (5)	C11—Ir—O1	95.02 (16)
C22—C17—C16	115.5 (4)	N1—Ir—O1	89.94 (14)
C19—C18—F3	116.3 (5)	N2—Ir—O1	93.88 (14)
C19—C18—C17	124.5 (5)	N3—Ir—O1	77.38 (14)
F3—C18—C17	119.2 (5)	C28—O1—Ir	116.0 (3)
C18—C19—C20	115.8 (5)		
